# Development of a Monoclonal Antibody to Pig CD69 Reveals Early Activation of T Cells in Pig after PRRSV and ASFV Infection

**DOI:** 10.3390/v14061343

**Published:** 2022-06-20

**Authors:** Yunfei Tian, Yuxin Hao, Maoli Dong, Shuai Li, Dongyue Wang, Fei Jiang, Qingqing Wang, Xiaoli Hao, Yi Yang, Nanhua Chen, Jianzhong Zhu, Junqing Guo, Jiajun Wu, Shaobin Shang, Jiyong Zhou

**Affiliations:** 1 Institute of Comparative Medicine, College of Veterinary Medicine, Yangzhou University, Yangzhou 225009, China; dz120200010@yzu.edu.cn (Y.T.); maoli.dong@legendbiotech.com (M.D.); lsmuzi98@163.com (S.L.); xlhao@yzu.edu.cn (X.H.); yangyi@yzu.edu.cn (Y.Y.); chnhlh@126.com (N.C.); jzzhu@yzu.edu.cn (J.Z.); 2 The Biosafety High-Level Laboratory Management Office, China Animal Disease Control Center, Beijing 102609, China; 18810121512@163.com (Y.H.); wangdongyuevip@126.com (D.W.); jiangfeiacuo@163.com (F.J.); wangqingq96@163.com (Q.W.); 3 Jiangsu Co-Innovation Center for Prevention and Control of Important Animal Infectious Diseases and Zoonosis, Yangzhou University, Yangzhou 225009, China; 4 Key Laboratory of Animal Immunology, Henan Academy of Agricultural Sciences, Zhengzhou 450008, China; 13838248132@163.com; 5 International Corporation Laboratory of Agriculture and Agricultural Products Safety, Yangzhou University, Yangzhou 225009, China; 6 College of Animal Science, Zhejiang University, Hangzhou 310058, China; jyzhou@zju.edu.cn

**Keywords:** porcine CD69, monoclonal antibody, T cell activation, PRRSV, ASFV

## Abstract

The CD69 molecule, as an early activation marker of lymphocytes, is often used to assess the activation of cellular immunity. However, for pigs, an anti-pig CD69 antibody is not yet available for this purpose after infection or vaccination. In this study, a monoclonal antibody (mAb) against pig CD69 was produced by peptide immunization and hybridoma technique. One mAb (5F12) showed good reactivity with pig CD69 that was expressed in transfected-HEK-293T cells and on mitogen-activated porcine peripheral blood mononuclear cells (PBMCs) by indirect immunofluorescence assay and flow cytometry. This mAb did not cross-react with activated lymphocytes from mouse, bovine, and chicken. Epitope mapping showed that the epitope recognized by this mAb was located at amino acid residues 147–161 of pig CD69. By conjugating with fluorochrome, this mAb was used to detect the early activation of lymphocytes in PRRSV- and ASFV-infected pigs by flow cytometry. The results showed that PRRSV infection induced the dominant activation of CD4 T cells in mediastinal lymph nodes and CD8 T cells in the spleen at 14 days post-infection, in terms of CD69 expression. In an experiment on ASFV infection, we found that ASFV infection resulted in the early activation of NK cells, B cells, and distinct T cell subsets with variable magnitude in PBMCs, spleen, and submandibular lymph nodes. Our study revealed an early event of lymphocyte and T cell activation after PRRSV and ASFV infections and provides an important immunological tool for the in-depth analysis of cellular immune response in pigs after infection or vaccination.

## 1. Introduction

The cluster of differentiation antigen 69 (CD69), recognized as the early activation surface marker of lymphocytes, is a type-II membrane protein of the C-type lectin superfamily [1] and is expressed on activated natural killer (NK) cells, macrophages, monocytes, granulocytes and B cells, and T cells in humans [2,3,4]. CD69 expression can be rapidly induced in vitro by stimulation with mitogens or TNF-α under experimental conditions [2]. Its expression was shown to be earlier than that of CD25 on NK cells and CD4 T cells in human peripheral blood mononuclear cells (PBMCs) upon allergen stimulation [5]. The upregulated expression of CD69 is thought to be indicative of the cytotoxic activity of NK cells, whereas CD25 expression implies proliferative potential [6]. In addition, the detection of CD69 is more sensitive than the detection of IFN-γ, in order to show T cell activation after mitogen stimulation [7]. Therefore, the detection of CD69 is a reliable indicator for the early activation of T cells. The activation state of immune cells after infection or immunization was routinely determined by detecting CD69 expression, along with other markers in human and mouse models [5,6,8,9,10,11]. For instance, by detecting CD69 expression, mycobacterium tuberculosis-specific CD4 T cells were shown to be activated in the mediastinal lymph node as early as on day 9 after infection [12] and tissue-resident memory CD8 T cells significantly increased in the skin in a model of vaccinia virus skin infection [13]. In addition, CD69 expression on CD4 T-cellT cells was found to be correlated with immune protection against tularemia [11]. However, these strategies have not yet been validated and applied in pigs, due to the lack of a specific antibody to pig CD69, hindering the evaluation of the early activation of cellular immunity in pigs during infection or immunization.

Pig CD69 consists of 200 amino acids (aa) with an intracellular region of 38 aa, a 26-aa transmembrane domain, and a 136-aa extracellular domain, and shares 75%, 67%, and 57% aa homology to bovine, human and murine CD69 molecules, respectively [14]. None of the commercially available anti-human or anti-mouse CD69 monoclonal antibodies (mAb) was found to cross-react with pig CD69 [15]. To date, the evaluation of porcine lymphocyte immune activation can be achieved by indirect methods, such as an IFN-γ enzyme-linked immunospot assay (ELISpot) and intracellular cytokine staining (ICS) [6,7,16,17]. These methods primarily detect the later activation of lymphocytes and are much less sensitive than directly detecting CD69 expression [7]. Although mAbs against porcine CD69 were reported [7], it remains to be validated for the detection of the early activation of immune cells in pigs, following infection and immunization.

Porcine reproductive and respiratory syndrome virus (PRRSV) and African swine fever virus (ASFV) are two of the most devastating pathogens that pose a tremendous threat to the pig industry worldwide. As the induction of a humoral immune response has not been very successful in the control of these two diseases, an understanding of cellular immunity against these viruses has become a research hotspot in order to dissect the mechanism of immune protection and develop more effective vaccines. Numerous studies showed that PRRSV infection or attenuated modified live virus (MLV) vaccination induced a relatively weak and delayed cellular immune response, where increased cytotoxic CD8 T cells and CD4^+^CD8^+^ T cells were detected at 4–5 weeks after infection or immunization [18,19], while IFN-γ-producing cells were occasionally detected as early as 2–3 weeks in individual animals after infection [20,21]. Although infection with the highly pathogenic PRRSV (HP PRRSV) strain was shown to cause the depletion/altered recruitment of Th cells, γδ T cells and B cells [22], IFN-γ-producing cells have been detected at 3 weeks post-infection [23]. Although the T cell epitopes recognized by pig CD8 T cells, γδ T cells, and CD4^+^CD8^+^ T cells were identified after PRRSV infection by an ICS and/or IFN-γ ELISpot assay [24,25], how soon these cells or other immune cells are activated after PRRSV infection or vaccination has not yet been addressed, in terms of CD69 expression. Recent studies also showed that highly virulent ASFV infection led to decreased T cell frequency, and no proliferation or T-bet-dependent activation of CD8 T cells were observed in domestic pigs [26], while the moderately virulent ASFV strain induced the proliferation of CD8α^+^ T cells, along with a loss of perforin expression at 5 days post-infection (dpi) [27]. There was no information given about the expression of the classic activation marker CD69 on each immune cell subset in the previous studies to demonstrate the early activation of cellular immunity after ASFV infection [26,27].

In the present study, we generated and validated a novel mAb against pig CD69 and identified the epitope recognized by this mAb. This mAb recognized pig CD69 expressed in transfected HEK-293T cells and on activated porcine PBMCs by flow cytometry. Using this mAb, in combination with flow cytometry, we detected the early activation of different lymphocyte subsets in pigs after PRRSV immunization and infection and ASFV infection.

## 2. Materials and Methods

### 2.1. Animals, Cells, and Virus Strains

Balb/c and ICR mice were purchased from the Center for Comparative Medicine of Yangzhou University. The SP2/0 and HEK-293T cell lines were held in our laboratory. A chimeric HP-PRRSV2 vaccine strain rJS-ORF2-6-CON and a virulent NADC30-like PRRSV type-2 strain SD17-36 were available from our previous study [28]. The ASFV HLJ/18 strain was isolated in 2018 as previously described [29], which was sequenced (GenBank accession number: MK333180) and belongs to genotype II ASFV [30], and was held in the China Animal Disease Prevention and Control Center. Pigs for the ASFV infection experiment were purchased from the Beijing Qingquanwan Pig Breeding Co., Ltd. (Beijing, China).

### 2.2. Porcine CD69 Polypeptide Immunization and Cell Fusion

The porcine CD69-peptide, consisting of 29 amino acids (residues 133–161) in the extracellular domain of porcine CD69, as well as three truncated peptides (Pep 1–3), with 1 extra cysteine at the N-terminus, are listed in Table 1 and were synthesized from Wuhan Dangang Biotechnology Co., Ltd. (Wuhan, China) and conjugated to KLH. Four 6-week-old female Balb/c mice were immunized intraperitoneally with 100 µg of KLH-conjugated CD69-peptide in Freund’s complete adjuvant (Sigma-Aldrich, Saint Louis, MO, USA). Two weeks later, a second immunization using an immunogen emulsified with Freund’s incomplete adjuvant was performed. The antibody titer against the CD69 peptide in peripheral blood was monitored. One week after the last immunization boost, the mice were euthanized. Blood was collected for the separation of anti-CD69 serum (polyclonal antibodies, pAb) and the spleens were harvested. Splenocytes were prepared and used to fuse with the mouse myeloma cell line, SP2/0. 

### 2.3. Hybridoma Screening, Ascites Production, Purification, and Fluorescence Labeling of CD69 mAb

The positive hybridoma screening was performed via ELISA and FCM. Positive hybridomas were cloned twice with a limiting dilution method. For ascites production, 5 × 10^5^ positive hybridomas were injected into a Balb/c mouse that had been pre-injected with 500 µL pristane. The ascites was extracted continuously, and the antibody was purified with an IgG Purification Kit (Beyotime, Shanghai, China), according to the manufacturer’s instructions. The Ig subclass and light-chain class of mAb were determined with a mouse immunoglobulin-isotyping ELISA kit (BD Pharmingen™, Franklin Lakes, NJ, USA) according to the manufacturer’s instructions. Purified mAb was labelled using DyLight^®^ 755 NHS Ester (Thermo Fisher, Waltham, MA, USA), as per the manufacturer’s instructions.

### 2.4. Cloning and Construction of pcDNA3.1-poCD69 Plasmid

Based on the pig CD69 gene sequence in NCBI (GenBank accession#: NM_214091), the full-length pig CD69 gene was amplified by RT-PCR from the porcine PBMCs that were pre-activated with PMA and ionomycin, with a forward primer: 5′-CCGGAATTC1ATGGGTTCTGAAAATTGTTCCACAACAG-3′ containing the restriction site EcoRI, along with a reverse primer: 5′-CCGCTCGAGTTAATGGTGAT GGTGATGATGTATGGAGGATTTACT-3′, containing the restriction site XhoI and a 6×His tag at the N-terminal. The PCR fragment was inserted into the pcDNA3.1 vector to construct recombinant plasmid pcDNA3.1-poCD69. Recombinant plasmids were confirmed by restriction enzymatic digestion and sequencing. The ultrapure plasmids were extracted using the QIAGEN Plasmid Midi Kit (QIAGEN, Frankfurt, Germany) for transfection.

### 2.5. Transfection and Indirect Immunofluorescence Assay

HEK-293T cells were grown on 6- or 96-well plates or coverslips and transfected with pcDNA3.1-poCD69 and the pcDNA3.1 vector using Lipofectamine^®^ 2000 DNA Transfection Reagent (Invitrogen, Carlsbad, CA, USA). At 24 h post-transfection, cells were washed twice with phosphate-buffered saline (PBS) and fixed with 4% paraformaldehyde in PBS for 20 min at 4 °C. The cells were washed three times with PBS and permeabilized with 0.5% Triton X-100 for 15 min. The coverslips were then incubated with anti-6×His tag mAb (1:1000) in PBS or anti-poCD69 mAb supernatant for 1 h. After three washes, the coverslips were incubated with Alexa Fluor 488-conjugated goat anti-mouse IgG antibody at room temperature (RT) for 1 h. Then, the coverslips were washed three times and treated with 4′,6′-diamidino-2-phenylindole (DAPI; Sigma-Aldrich, Saint Louis, MO, USA). After five washes, the coverslips were observed under an Olympus BX51 inverted fluorescence microscope (Olympus, Tokyo, Japan).

### 2.6. Western Blotting

The plasmid-transfected cells were harvested in cell lysis buffer, supplemented with 1% PMSF (Beyotime, Shanghai, China). The cell lysates were resolved on 12% SDS-PAGE gels and transferred onto nitrocellulose membranes (Merck, Darmstadt, Germany). The membrane was blocked with 5% skimmed milk in Tris-buffered saline containing 0.2% Tween-20 (TBST) at RT, followed by incubation with anti-poCD69 mAb supernatant or anti-6×His tag mAb for 1 h at RT. After washing 5 times, horseradish peroxidase (HRP)-conjugated goat anti-mouse IgG (H + L) antibody (Bioworld Technology, Dublin, OH, USA) was added and incubated for another 1 h at RT. After washing 5 times, the specific bands were developed using enhanced chemiluminescence (ECL) reagents, NcmECL Ultra (NCM Biotech, Suzhou, China), and visualized on Bio-Rad GelDoc XR+ (Bio-Rad, Hercules, CA, USA).

### 2.7. Dot-ELISA

The nitrocellulose membrane was dotted with 1μg of each peptide, conjugated with KLH, and dried for 10 min at RT, followed by blocking with 5% skimmed milk in PBS for 2 h at 37 °C. The membrane was washed thoroughly with 0.05% PBST and incubated with diluted mAb supernatant for 1 h at RT. After washing 5 times with PBST, the membrane was incubated with HRP-conjugated goat anti-mouse IgG (H + L) antibody, diluted at 1:5000 for 1 h at RT. After washing 5 times again, the color was developed with TMB substrate (Mabtech, Stockholm, Sweden).

### 2.8. Infection Experiments with PRRSV and ASFV

For the PRRSV immunization and infection experiment, we took advantage of the samples collected from a previous trial experiment [28]. Briefly, ten 4-week-old PRRSV-free piglets were equally divided into two groups. One group of pigs was first inoculated with 1 mL 10^5.0^ TCID_50_/mL rJS-ORF2-6-CON intramuscularly, into the triangle area of the neck, and the remaining 1 mL was slowly administered droplets to the pig’s nostril to ensure full absorption. The other group was inoculated with an equal volume of RPMI-1640 medium as a control. At 42 dpi, both groups were challenged with 2 mL of virulent PRRSV NADC30-like SD17-38 isolate at a titer of 10^5.0^ TCID_50_. While the clinical score and rectal temperatures were measured each day for the previous study, the peripheral blood of each animal was collected on days 7 and 14 post-challenge (dpc), and mediastinal lymph nodes and spleens from survival pigs were harvested at 14 dpc for the detection of CD69 expression on each immune cell subset by flow cytometry.

For the ASFV infection experiment, six 2-month-old healthy piglets without ASFV were randomly divided into two groups. One group was challenged with a 10 TCID_50_ dose of the ASFV HLJ/18 strain by intramuscular injection, while another group was mock-infected by injecting sterile PBS. The peripheral blood, spleens, and submandibular lymph nodes were partially collected on days 5 and 7 after infection, for the detection of CD69 expression by flow cytometry.

### 2.9. Single-Cell Suspension Preparation and Culture

Single-cell suspensions from peripheral blood and tissues were prepared as in a previous study [31]. Briefly, peripheral blood mononuclear cells (PBMCs) were separated from heparinized peripheral blood of pigs by gradient density centrifugation with a Porcine PBMC isolation KIT (Haoyang Co., Ltd., Tianjin, China). Single-cell suspensions from the lymph node and spleen were prepared by grinding the lymph nodes and spleen through a 70-μm cell strainer (BD, Biosciences, Franklin Lakes, NJ, USA) in 2% FBS RPMI-1640 medium. The red blood cells in the samples were lysed with red blood-cell lysis buffer (Solarbio, Beijing, China). The cell viability of each sample was checked with trypan blue staining and the live cells were counted using a hemocytometer; the final cell concentration was adjusted to 2 × 10^7^ cells/mL.

For the in vitro activation of lymphocytes, 2 × 10^6^ PBMCs were plated in triplicate on 96-well round-bottom plates in 200 μL of complete RPMI-1640 medium containing 10% fetal bovine serum (Sigma-Aldrich, Saint Louis, MO, USA), 100 U/mL penicillin, and 100 mg/mL streptomycin (Gibco, New York, NY, USA), then stimulated with or without PMA and ionomycin for 6 h at a final concentration of 50 ng/mL and 500 ng/mL, respectively. Thereafter, cells were harvested for testing the CD69 expression by flow cytometry.

### 2.10. Flow Cytometry

The single-cell suspensions from peripheral blood, spleens, and lymph nodes were plated in 96-well V-bottom plates, with 2×10^6^ cells in each well, in 100 μL FACS buffer (0.5% BSA PBS). After centrifugation, the cells were incubated, either with hybridoma supernatant followed by staining with Alexa Fluor 488-labelled goat anti-mouse-IgG antibody, or were directly stained with 50 μL of an antibody cocktail containing Dylight^®^755-conjugated anti-poCD69 mAb (Dylight^®^755-CD69) and anti-porcine CD3, CD8α, CD4, TCRγδ, CD21 SLA-II DR, CD163, and CD172a antibodies with different fluorochrome for 20 min at RT, as previously reported [27,32]. A list of the antibodies used in the FACS is shown in Table 2. In brief, for the detection of CD69 expression on different leukocyte subsets, the cells were stained with 50 μL of antibody cocktail containing Dylight^®^755-CD69, anti-porcine CD3, CD8α, CD4, and CD21, or a cocktail containing Dylight^®^755-CD69, SLA-II DR, CD163, and CD172a. For the PRRSV experiment, cells were stained with 50 μL of an antibody cocktail containing Dylight^®^755-CD69, anti-porcine CD3, and CD4, while for the ASFV experiment, cells were stained with 50 μL of an antibody cocktail containing Dylight^®^755-CD69, anti-porcine CD3, CD8α, CD4, TCRγδ, and CD21. The cells were then washed once with FACS buffer by spinning them down at 400 g for 5 min at 4 °C. A minimum number of 100,000 cells was acquired for FACS analysis. Flow cytometry was performed with a FACS LSR Fortessa (BD Biosciences, Franklin Lakes, NJ, USA) and the data were analyzed using FlowJo software (Tree Star Inc., Ashland, OR, USA).

### 2.11. Statistical Analysis

Statistical analysis was performed using the GraphPad Prism software (GraphPad, La Jolla, CA, USA). When comparing experimental values from two groups, one- or two-tailed Student’s *t*-tests were routinely used. The statistical significance was noted (*** *p* < 0.001; ** *p* < 0.01; * *p* < 0.05).

## 3. Results

### 3.1. Generation and Characterization of Monoclonal Antibodies against Pig CD69

**Figure 1:**
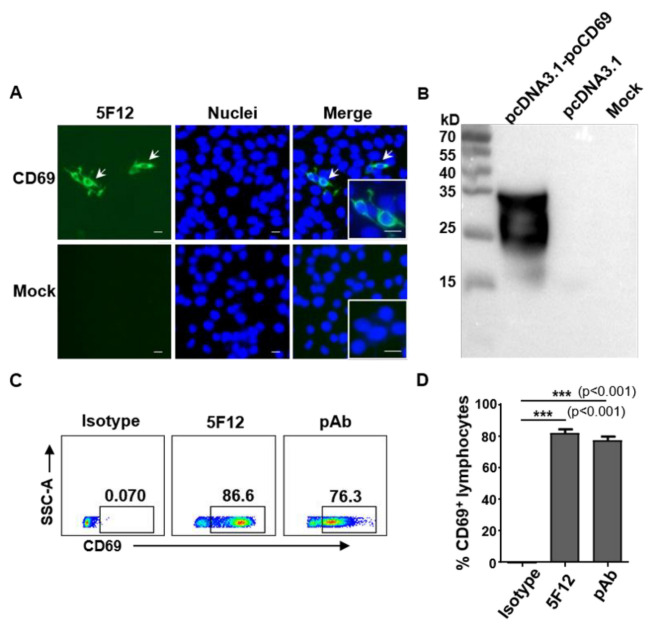
mAb 5F12 specifically recognizes recombinant and natural porcine CD69 protein. (**A**) Recombinant porcine CD69, expressed in pcDNA3.1-poCD69-transfected HEK-293T cells, was detected by mAb 5F12 against porcine CD69. The CD69-positive cells (white arrows) and negative cells were magnified. Scale bars, 40 μm. (**B**) mAb 5F12 specifically recognizes recombinant porcine CD69 in the cell lysates of pcDNA3.1-poCD69-transfected HEK-293T cells but not in pcDNA3.1-transfected or un-transfected HEK-293T cells (Mock) by Western blotting. (**C**,**D**) The expression of CD69 on pig lymphocytes was detected by mAb 5F12 and polyclonal anti-poCD69 (pAb) antibodies, followed by Alexa Fluor 488-labeled goat anti-mouse-IgG antibody, via flow cytometry after stimulation with PMA (50 ng/mL) and ionomycin (500 ng/mL) for 6 h. These CD69^+^ lymphocytes were gated on lymphocytes within the PBMCs of pigs. *** *p* < 0.001.

One monoclonal antibody against pig CD69 (mAb 5F12) was generated in this study. The mAb 5F12 strongly reacted with KLH-conjugated CD69 peptide but not with unconjugated KLH in the ELISA, with a titer of 1:25600. The Ig subclass of 5F12 was IgG1, with a κ light chain. In order to examine their reactivity with pig CD69 protein, HEK-293T cells were transfected with the pcDNA3.1-poCD69 and pcDNA3.1 plasmids, respectively. The reactivity of mAb 5F12 with eukaryotically expressed pig CD69 was examined by indirect immunofluorescence assay (IFA) and Western blotting. As shown in Figure 1, 5F12 specifically reacted with the recombinant pig CD69 expressed in pcDNA3.1-poCD69-transfected 293T cells by IFA (Figure 1A). The Western blotting showed that 5F12 recognized two bands with molecular weights of between 25 and 35 kD in the cell lysates of pcDNA3.1-poCD69-transfected 293T cells but not in the cell lysates of pcDNA3.1-transfected or mock-transfected cells (Figure 1B), which may indicate two monomers that were disrupted by SDS-PAGE, as CD69 is a 60 kD type-II membrane protein composed of a 27/33 kD disulfide-linked homodimer [2]. These results indicate that mAb 5F12 can specifically recognize recombinant pig CD69 protein. To confirm whether mAb 5F12 recognizes natural CD69 expressed by pig lymphocytes, PBMCs from healthy pigs were stimulated with PMA/ionomycin and stained with mAb 5F12, mouse anti-pig CD69 polyclonal antibody (pAb), and mouse isotype IgG1, respectively, followed by Alexa Fluor^®^488-labelled secondary anti-mouse IgG antibody and were then examined by flow cytometry. As shown in Figure 1C,D, 81.7 ± 4.95% and 77.2 ± 4.61% of activated PBMCs were stained positive by 5F12 and CD69 pAb but not by the isotype IgG1, suggesting that mAb 5F12 specifically recognizes the natural CD69 expressed by activated lymphocytes.

### 3.2. Anti-Pig CD69 mAb 5F12 Recognizes Epitope Residues 147–161 of Pig CD69

**Figure 2:**
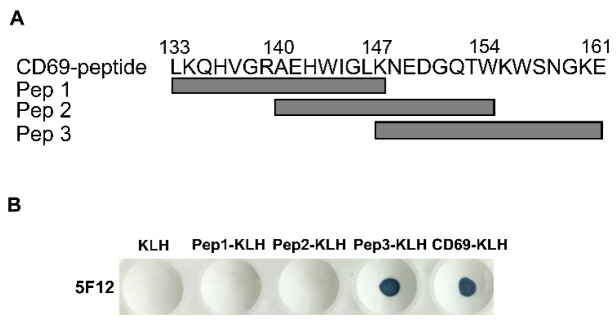
The identification of the epitope recognized by mAb 5F12 against CD69 by dot-ELISA. (**A**) Three overlapping peptides covering the CD69 polypeptide (aa 133–161) were synthesized and named Pep 1 (aa 133–147), Pep 2 (aa 140–154), Pep 3 (aa 147–161), an extra cysteine was added at the N terminus of each peptide for the conjugation of KLH. (**B**) These peptides were conjugated to KLH carrier and their reactivity with anti-poCD69 mAb 5F12 was determined by dot-ELISA.

In order to identify the epitope recognized by mAb 5F12, three 15-mer overlapping peptides covering the antigenic polypeptides 133–161 of pig CD69 were synthesized and named Pep 1 (aa 133–147), Pep 2 (aa 140–154), and Pep 3 (aa 147–161), respectively (Figure 2A). These peptides were conjugated with KLH and were used to test their reactivity with 5F12 via dot-ELISA. As shown in Figure 2B, 5F12 specifically recognized Pep 3 and the whole antigenic polypeptide, but not the empty carrier KLH. These results suggest that the epitope recognized by mAb 5F12 is located at aa 147–161 of the pig CD69 protein.

### 3.3. mAb 5F12 Shows Weak or No Cross-Reactivity with PBMCs from Bovine, Mice, and Chickens

**Figure 3:**
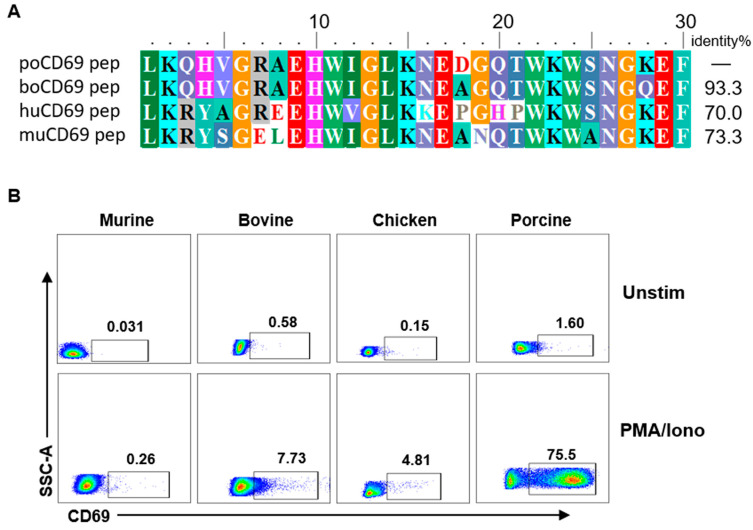
Anti-poCD69 mAb 5F12 shows weak or no cross-reactivity with bovine, mouse, and chicken CD69. (**A**) Homologous comparison of CD69 polypeptide (aa 133–161) in pig samples and human, mouse, and bovine samples. The percentage of homology was indicated. (**B**) The cross-reactivities of Dylight^®^755-conjugated anti-poCD69 mAb 5F12 with PBMCs from mice, cows, and chickens were examined by flow cytometry after the in vitro stimulation of PBMCs with or without PMA (50 ng/mL) and ionomycin (500 ng/mL) for 6 h (Unstim vs. PMA/Iono). The CD69^+^ lymphocytes were gated on lymphocytes within PBMCs. The data shown are representative of three independent experiments.

Anti-human or anti-mouse CD69 mAbs are widely used to evaluate the early activation of lymphocytes in human and mouse studies, but they are not cross-reactive with porcine CD69 [15]. We wondered whether 5F12 cross-reacts with bovine, murine, and chicken CD69s. We firstly compared the amino acid identities of the polypeptide of CD69 (aa 133–161) between pig and human, mouse, and bovine. The result showed that the pig shared 93.3%, 70.0% and 73.3% amino acid homology with bovine, human, and murine in the region of 133–161 of CD69, respectively (Figure 3A), implying the potential cross-reactivity of 5F12 with bovine CD69. To confirm this, the reactivities of 5F12 with the activated PBMCs from mouse, bovine, chicken, and pig were compared by flow cytometry. The results showed that mAb 5F12 strongly reacted with pig lymphocytes but showed slight or no reactivity with lymphocytes from mice, bovine, and chickens (Figure 3B), suggesting no cross-reactivity of mAb 5F12 with mouse, bovine, and chicken CD69s.

### 3.4. Detection of CD69 Expression on Different Leukocyte Subsets by Flow Cytometry

**Figure 4:**
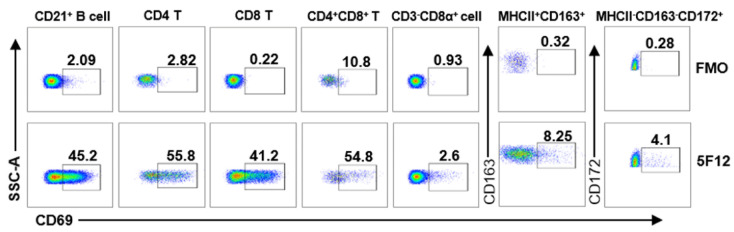
Anti-poCD69 mAb 5F12 is valid for the detection of CD69 expression on different leukocyte subsets of pigs. PBMCs from healthy pigs were stimulated with PMA (50 ng/mL) and ionomycin (500 ng/mL) for 6 h, then stained with a cocktail of antibodies containing Dylight^®^755-CD69, anti-porcine CD3, CD8α, CD4, and CD21 or a cocktail containing Dylight^®^755-CD69, SLA-II DR, CD163, and CD172a. The expression of CD69 on CD21^+^ B cells (gated on lymphocytes) and T cell subsets (CD4^+^, CD8^+^, CD4^+^CD8^+^, gated on CD3^+^ T lymphocytes), NK cells (gated on CD21^−^CD3^−^CD8α^+^ cells), monocytes/macrophages (MHCII^+^CD163^+^), and neutrophils (MHCII^−^CD163^−^CD172^+^) was determined by flow cytometry. Monocytes/macrophages and neutrophils were gated on leukocytes, excluding lymphocytes.

CD69, as an early activation marker, is expressed on activated B cells, T cells, NK cells, macrophages, monocytes, and granulocytes [4,33]. In order to test and validate if mAb 5F12 can be used to detect CD69 expression on different leukocyte subsets by flow cytometry, mAb 5F12 was purified and conjugated with fluorochrome DyLight^®^755 and then used to directly stain pig PBMCs after stimulation with PMA/ionomycin, along with a group of antibodies to define each subset, as previously reported [27,32]. As shown in Figure 4 and Figure S1, CD69 is dominantly expressed on CD21^+^ B cells (45.8 ± 2.65%) and T cell subsets, including CD4^+^ (55.83 ± 2.25%), CD8^+^ (42.6 ± 2.86%) and CD4^+^CD8^+^ T cells (53.1 ± 3.29%) but is only expressed slightly on NK cells (CD3^−^CD8α^+^), monocytes/macrophages (MHCII^+^CD163^+^), and neutrophils (MHCII^−27^CD163^−^CD172^+^) upon activation with PMA/ionomycin. These results indicate that mAb 5F12 can be used to detect CD69 expression on the activated lymphocytes of pigs using multicolor flow cytometry.

### 3.5. Detection of CD69 Expression on Lymphocytes in Pigs after Challenge with PRRSV

**Figure 5:**
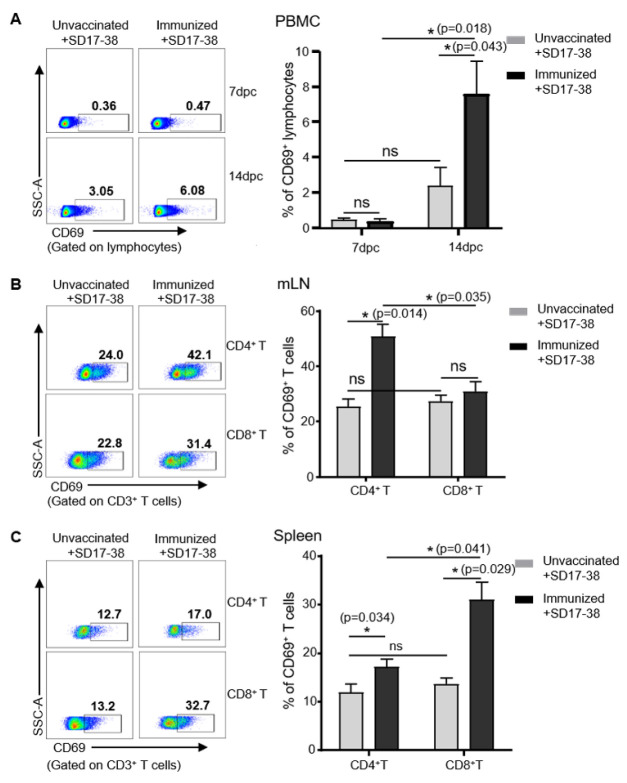
The detection of CD69 expression on T cells in different organs after PRRSV immunization and infection. PRRSV-free piglets were immunized intranasally and intramuscularly with the rJS-ORF2-6-CON vaccine or were administered RPMI-1640 (as the control), then challenged with a virulent PRRSV NADC30-like SD17-38 isolate on day 42 post-immunization. Peripheral blood, mediastinal lymph nodes (mLN), and spleens were harvested on days 7 and/or 14 post-challenge (dpc) and single-cell suspensions were prepared for the detection of CD69 expression on T cell subsets via flow cytometry. (**A**) Representative pseudocolor plots (left panel) and the dynamic changes (right panel) of total CD69^+^ lymphocytes in the PBMCs of unvaccinated (*n* = 3) and immunized pigs (*n* = 3) after challenge. (**B**,**C**) Representative dot-plots (left panel) and the comparison (right panel) of CD69^+^CD4^+^ and CD69^+^CD8^+^ T cells in the mLNs and spleens of unvaccinated (*n* = 3) and immunized pigs (*n* = 3) at 14 dpc. Data shown are mean ± SEM. ns, no statistical significance. * *p* < 0.05.

In order to validate the use of 5F12 for detecting the early activation of lymphocytes during vaccination or infection, a model of PRRSV infection and vaccination was employed in which piglets were immunized with or without chimeric PRRSV type 2 vaccine rJS-ORF2-6-CON and then challenged six weeks later with a virulent NADC30-like SD17-38 isolate, as previously reported [28]. PBMCs from the unimmunized and immunized pigs were isolated on days 7 and 14 dpc for the detection of CD69 by flow cytometry. As shown in Figure 5A, there were very few CD69^+^ lymphocytes (less than 0.5%) in both groups at 7 dpc. However, at 14 dpc, the immunized and challenged pigs had 7.59 ± 3.23% CD69^+^ of lymphocytes within PBMCs, while the unvaccinated and challenged pigs had only 2.08 ± 1.1% CD69^+^ of lymphocytes, indicating that the PRRSV challenge induced more activated lymphocytes in the immunized pigs at 14 dpc, compared to the control. Further examination of CD69 expression on T cells from mediastinal lymph nodes (mLN) and spleens showed that the unvaccinated and challenged pigs had similar numbers of CD69^+^CD8^+^ and CD69^+^CD4^+^ T cells in the mLNs (27.2 ± 3.6% vs. 25.36 ± 4.56%) and spleens (13.56 ± 2.17% vs. 11.93 ± 2.56%) at 14 dpc (Figure 5B,C). In contrast, the immunized and challenged pigs had more CD69^+^CD4^+^ T cells (49.63 ± 6.82%) than CD69^+^CD8^+^ T cells (30.6 ± 3.86%) in the mLN and more CD69^+^CD8^+^ T cells (30.96 ± 4.33%) than CD69^+^CD4^+^ T cells (17.13 ± 1.26%) in the spleens at the same time point (Figure 5B,C). These results indicate that PRRSV infection induced the early activation of lymphocytes, and specifically of T cells, in pigs and different numbers of CD69-positive CD4 and CD8 T cells in the mLNs and spleens of immunized pigs after the challenge.

### 3.6. Detection of CD69 Expression on Different Lymphocyte Subsets in Pigs after ASFV Infection

**Figure 6:**
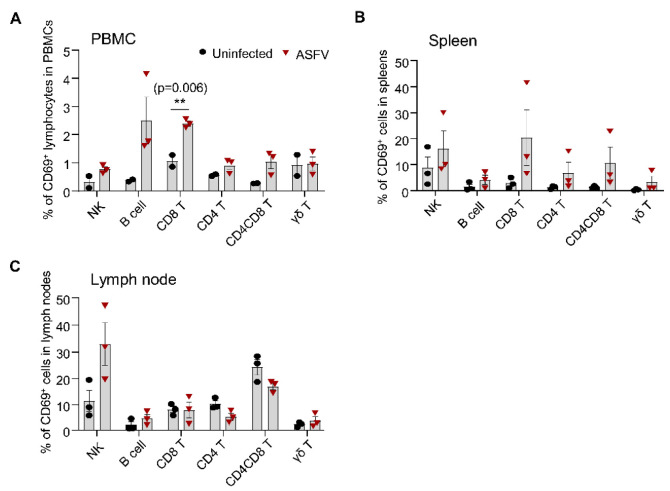
ASFV infection induces the early activation of different lymphocyte subsets in the blood and lymphoid organs of pigs. Healthy piglets were infected or not infected with the ASFV HLJ/18 strain by intramuscular injection. Peripheral blood, spleens, and submandibular lymph nodes were collected on day 5 after infection and single-cell suspensions were prepared for the detection of CD69 expression on different lymphocyte subsets, using flow cytometry. The percentages of CD69-positive NK cells, B cells, CD8, CD4, and CD4^+^CD8^+^ T cells, as well as γδ T cells in the PBMCs (**A**), spleen (**B**), and submandibular lymph node cells (**C**) were compared between mock-infected and ASFV-infected pigs (*n* = 3). Data shown are mean ± SEM. ns = no statistical significance. ** *p* < 0.01.

ASFV infection causes a highly lethal acute hemorrhagic disease with near 100% mortality in domestic pigs [29]. The infected pigs usually die 7–14 days after infection. It is unclear whether such an acute infection with ASFV leads to the transient or early activation of immune cells in the blood and lymphoid organs. We used CD69 as a readout to assess the early activation of immune cells after infection with a genotype II ASFV strain HLJ/18. As shown in Figure 6 and Figure S2, ASFV infection induced the significantly upregulated expression of CD69 on CD8^+^ T cells and a trend toward an increase in the expression of CD69 on NK cells, B cells, and CD4^+^ and CD4^+^CD8^+^ T cells but not on γδ T cells in the blood at 5 dpc, compared to the control, although the percentage of CD69-positive cells was low (below 5%) (Figure 6A). The magnitude of CD69 expression in the spleens and lymph nodes was higher than that in the blood on all the subsets we examined after ASFV HLJ/18 infection (Figure 6B,C). However, only a trend toward an increase in the expression of CD69 on CD8^+^, CD4^+^, and CD4^+^CD8^+^ T cells and γδ T cells but not on NK and B cells was observed in the spleens of the infected piglets, compared to the control. There was no significant difference between the two groups, probably due to the huge individual variability in the infected animals (Figure 6B). In the lymph nodes, the NK cells showed a marked increase in the expression of CD69 (30.03 ± 13.58%) but there was no increase or even a decrease in CD69 expression on other subsets after ASFV infection (Figure 6C). These results suggested that ASFV infection induced the early activation of immune cells and that the magnitude of activation varied in different lymphocyte subsets and different organs, in terms of CD69 expression.

## 4. Discussion

Cellular immunity plays a central role in the control of bacteria and virus infection. There have been many studies focusing on cellular immune responses to devastating swine diseases such as the swine influenza A virus (SIV), PRRSV, and ASFV [24,25,26,27,34]. Although the dynamic changes of distinct immune cell subsets, cytotoxic T cells, and T cell epitopes have been examined after infection or immunization in some studies [24,25,26,27,34], the early activation of immune cells, a critical event in the development of adaptive immunity, have never been revealed in pigs. CD69 is well recognized as a very early activation marker in human and mouse studies [2,35], but its application has not yet been validated in pigs in the setting of viral infections. In this study, we generated and validated a mAb against pig CD69 that recognizes the epitope aa 147–161. Using this mAb, we detected the earlier activation of lymphocytes than has previously been reported in pigs after PRRSV immunization and infection. In addition, we found that ASFV acute infection induced the early activation of distinct lymphocyte and T cell subsets with different magnitudes in different organs, in terms of CD69 expression.

As CD69 is indicative of earlier activation than IFN-γ secretion, CD25 expression, or proliferation [5,7], an anti-CD69 antibody is also desirable for the evaluation of cellular immunity in other species, such as cows and chickens. Therefore, we tested the cross-reactivity of mAb 5F12 with other species. However, we did not find significant cross-reactivity of mAb 5F12 with bovine, mouse, and chicken CD69s. Notably, bovine and pig have the highest identity in the epitope 147–161 of CD69, with only two amino acids being different. We tried to truncate either the N-terminal or C-terminal amino acids of the epitope to identify the critical amino acid for recognition by 5F12 but failed. In addition, as the CD69 ortholog is not yet identified in the chicken genome, we were not able to compare the homology of porcine and chicken CD69. Although CD69 was reported to be expressed on activated B cells, T cells, NK cells, monocytes, macrophages, and granulocytes [4,33], our results showed that CD69 is readily detected on activated B cells and distinct T cell subsets but just slightly or barely detectable on NK cells, macrophages, and granulocytes in pigs after mitogen stimulation (Figure 4).

Although anti-porcine CD69 mAbs were generated in this and in another study [7], whether they are applicable for detecting the early activation of lymphocytes in the setting of viral infection has not yet been proved. Using the PRRSV and ASFV infection models, we demonstrated that the mAb 5F12 that we generated can be used to detect the early activation of lymphocytes in pigs by flow cytometry, after infection and immunization. It is well known that PRRSV infection or MLV vaccination induces a weak and delayed cellular immune response. The activation of cell-mediated immunity was generally detected at 3–4 weeks after PRRSV infection, in terms of virus-specific lymphoproliferation, IFN-γ production, and cytotoxic CD8 T cells [18,20,24,36,37]. However, by detecting IFN-γ-secreting cells via an ELISpot assay, the cellular immunity was shown to be activated as early as 2–3 weeks in individual pigs after PRRSV infection [20,21] and can be detected at 17 dpc in the previously immunized and challenged pigs [23]. In this study, by directly detecting CD69 expression, we identified that the lymphocytes in PBMCs were activated as early as 14 dpc in the PRRSV-infected pigs (2.08 ± 1.1%) and the immunized and challenged pigs (7.59 ± 3.23%) (Figure 5A), showing the advantage of CD69 regarding the early detection of lymphocyte activation. Of note, CD69 expression is very low at a steady state or at 7 days after PRRSV infection in the PBMCs of pigs, whereas its expression is much higher in the spleen and lymph node than in PBMCs after PRRSV infection (e.g., 14 dpc) (Figure 5B,C). This phenomenon indicates that the upregulated expression of CD69 on T cells may preferentially represent the local and resident activation of T cells. Indeed, a previous study has shown that CD69 binds to the G-protein-coupled sphingosine 1-phosphate receptor-1 (S1P1) and inhibits S1P1-mediated lymphocyte egress from lymph nodes [38]. A recent study also confirmed that CD69 was almost undetectable in blood but was highly expressed on tissue-resident memory T cells in the local tissues of pigs after SIV infection [39].

Similarly, we also proved that ASFV infection induced the early activation of different lymphocyte subsets. Unlike a previous report that no activation of CD8 T cells was detected in domestic pigs after infection with the highly virulent ASFV strain Armenia08 in terms of Ki67 expression, which is an indicator of proliferation, and T-bet upregulation in the blood and lymphoid organs [26], we detected the apparent upregulation of CD69 on NK cells, B cells, and T cells in the blood, on distinct T cell subsets in the spleen, and on NK cells in the LNs at 5 dpi, showing the early activation of lymphocytes after ASFV infection. We also detected IFN-γ and TNF-α production by T cells ex vivo, via intracellular cytokine staining, from 3 to 7 dpi but detected no increase in the frequency of IFN-γ- and TNF-α-positive T cells (data not shown), suggesting that CD69 is a more sensitive marker for detecting the early activation of lymphocytes. However, it is not clear whether the activation is virus-specific or not, as the pro-inflammatory cytokines induced by ASFV may activate lymphocytes via the bystander pathway [40]. Surprisingly, although the frequency of γδ T cells is high in pigs [41] and they are prone to activation by proinflammatory cytokines [42], there was a very low proportion of activated γδ T cells in the spleen, blood, and LNs, which is consistent with the findings of a previous report [27]. Although our data showed that ASFV infection induced the activation of lymphocytes, based on CD69 expression, this activation may be transient, as ASFV infection causes the rapid progression of the disease and the subsequent death of pigs. How ASFV eventually impairs or inhibits the development of adaptive immunity needs further investigation.

In summary, we generated and validated mAb 5F12 against porcine CD69; it recognizes the epitope 147–161 and has no cross-reactivity with other species. This mAb can be used to detect the activation of distinct lymphocyte subsets via flow cytometry upon mitogen stimulation or infection with PRRSV and ASFV. Using this approach, we detected the earlier activation of lymphocytes than previously reported in pigs after PRRSV immunization and infection and found that ASFV infection induced the early activation of distinct lymphocyte subsets, with different magnitudes. Our study reveals an early event of lymphocytes and T cell activation after PRRSV and ASFV infections and provides an important immunological tool for the in-depth analysis of cellular immune response in pigs after infection or vaccination.

## Figures and Tables

**Table 1:** The peptides synthesized in the experiment.

Peptides	Sequence	Start	End	Length *
CD69-peptide	CLKQHVGRAEHWIGLKNEDGQTWKWSNGKE	133	161	30
Pep 1	CLKQHVGRAEHWIGLK	133	147	16
Pep 2	CAEHWIGLKNEDGQTW	140	154	16
Pep 3	CKNEDGQTWKWSNGKE	147	161	16

* indicates that an extra cysteine was added at the N terminus of each peptide for the subsequent conjugation of KLH.

**Table 2:** Antibodies used in this study.

Antigen	Clone	Isotype	Conjugate	Source
CD3	BB23-8E6-8C8	Mouse IgG2a	PerCP-cy5.5	BD
CD4	74-12-4	Mouse IgG2b	PE-Cy™7	BD Pharmingen
CD8α	76-2-11	Mouse IgG2a	biotin	Southernbiotech
CD21	BB6-11C9.6	Mouse IgG1	AlexaFluor^®^488	Southernbiotech
γδTCR	MAC320	Rat PVG IgG2a	PE	BD
CD69	5F12	Mouse IgG1	Dylight^®^755	in-house
CD163	2A10/11	Mouse IgG1	RPE	Bio-rad
CD172a	74-22-15	Mouse IgG1	FITC	Southernbiotech
SLA II DR	2E9/13	Mouse IgG2b	APC	Bio-rad
Mouse IgG	Poly4053	Goat polyclonal IgG	AlexaFluor^®^488	Biolegend
Streptavidin	—	—	BV 510™	Biolegend

## Data Availability

Not applicable.

## Supplementary Materials

The following are available online at https://www.mdpi.com/article/10.3390/v14061343/s1, Figure S1: Gating strategy of lymphocyte subsets, monocytes/macrophages and neutrophils in flow cytometry analysis; Figure S2: Representative zebra-plots of CD69^+^ lymphocyte subsets after ASFV infection.
