# Supplementary material for: Development of a Monoclonal Antibody to Pig CD69 Reveals Early Activation of T Cells in Pig after PRRSV and ASFV Infection
Source: Viruses. 2022 Jun 20;14(6):1343. doi: 10.3390/v14061343 (PMC9231377; doi:10.3390/v14061343)
Supplement: Supplementary file 1 [file viruses-14-01343-s001.zip › viruses-1719373-supplementary.pdf]

Supplementary Files

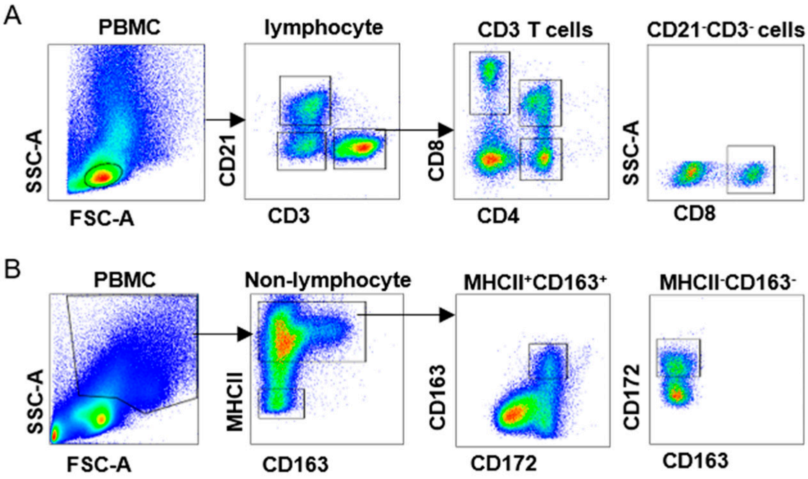

Figure S1. Gating strategy of lymphocyte subsets, monocytes/macrophages and neutrophils in flow cytometry analysis. (A) $CD21^+$ B cells are gated on lymphocytes. T cell subsets (CD4 T, CD8 T, $CD4^+CD8^+$ T) are gated on $CD3^+$ T lymphocytes. NK cells ($CD3^-CD8\alpha^+$) are gated on $CD21^-CD3^-CD8\alpha^+$ lymphocytes. (B) Macrophages ($MHCII^+CD163^+CD172a^+$) are gated on leukocytes excluding lymphocytes. Neutrophils ($MHCII^-CD163^-CD172^+$) are gated on $MHCII^-CD163^-CD172^+$ leukocytes excluding lymphocytes.

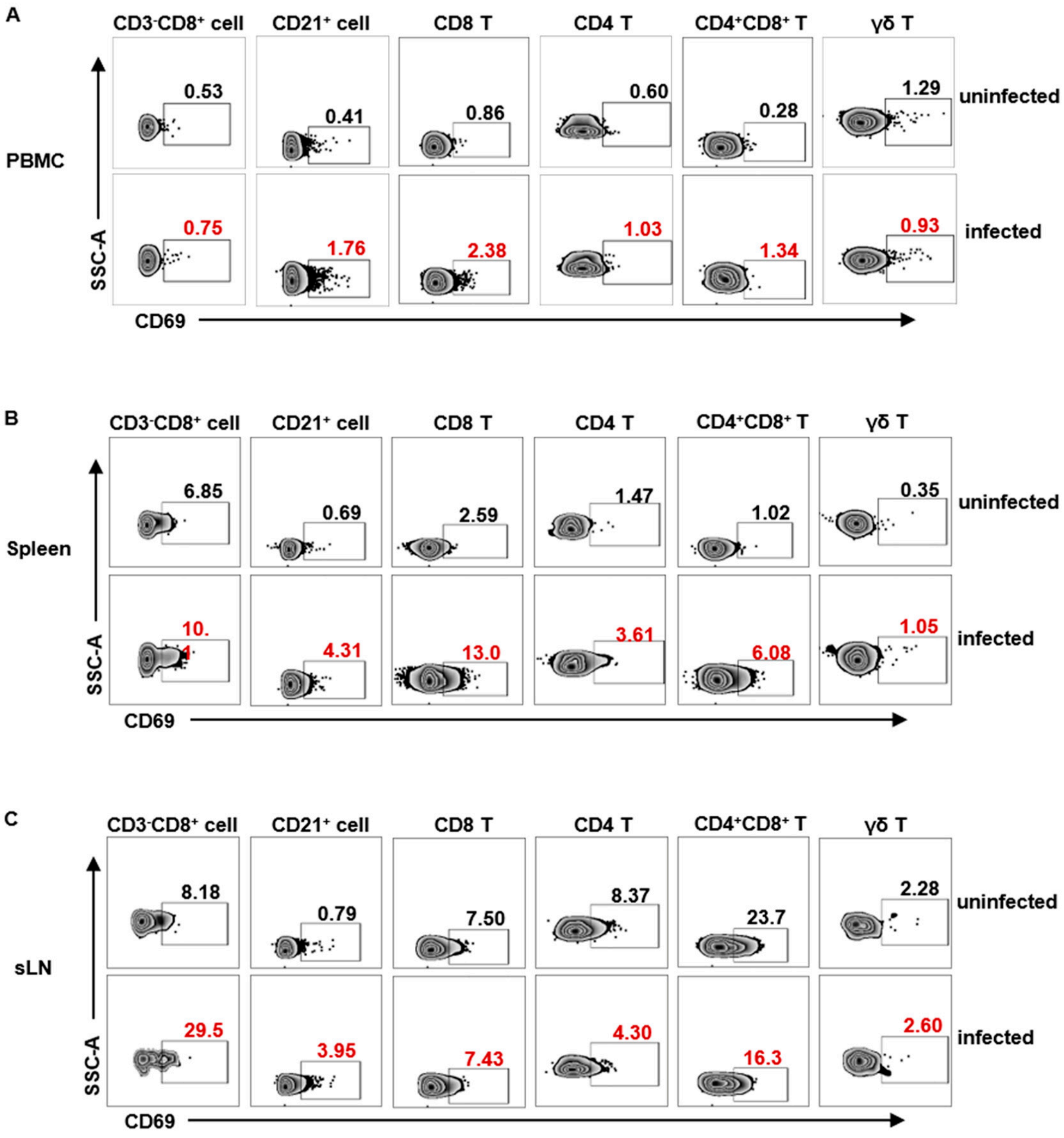

Figure S2 Representative zebra-plots of CD69$^+$ lymphocyte subsets after ASFV infection.

Healthy piglets were infected or uninfected with ASFV HLJ/18 strain by intramuscular injection. Peripheral blood, spleens and submandibular lymph nodes were collected on day 5 after infection and single cell suspension was prepared and stained with antibody cocktail (anti-pig CD69, CD21, CD3, CD8, CD4 and $\gamma\delta$TCR). Pig CD69 expression on distinct lymphocyte subsets from PBMCs (A), spleen (B) and submandibular lymph node (sLN) (C) of uninfected and infected piglets were examined by flow cytometry. NK cells (CD3$^-$CD8α^+) were gated on CD21$^-$CD3$^-$CD8α^+ lymphocytes. B cells (CD21$^+$) and $\gamma\delta$ T cells (TCR$\gamma\delta^+$) were gated on lymphocytes. T cell subsets (CD8 T, CD4 T, CD4$^+$CD8$^+$ T) were gated on CD3$^+$ lymphocytes.
